# Prediction of surface roughness based on fused features and ISSA-DBN in milling of die steel P20

**DOI:** 10.1038/s41598-023-42968-4

**Published:** 2023-09-24

**Authors:** Miaoxian Guo, Jin Zhou, Xing Li, Zhijian Lin, Weicheng Guo

**Affiliations:** 1 College of Mechanical Engineering, University of Shanghai for Science and Technology, Shanghai, 200093 China; 2 Beijing Spacecrafts Co. Ltd., Beijing, 100094 China; 3 Aplos Machines Manufacturing (Shanghai) Co. Ltd., Shanghai, 201306 China

**Keywords:** Engineering, Mechanical engineering

## Abstract

The roughness of the part surface is one of the most crucial standards for evaluating machining quality due to its relationship with service performance. For a preferable comprehension of the evolution of surface roughness, this study proposes a novel surface roughness prediction model on the basis of the unity of fuse d signal features and deep learning architecture. The force and vibration signals produced in the milling of P20 die steel are collected, and time and frequency domain feature from the acquired signals are extracted by variational modal decomposition. The GA-MI algorithm is taken to select the signal features that are relevant to the surface roughness of the workpiece. The optimal feature subset is analyzed and used as the input of the prediction model. DBN is adopted to estimate the surface roughness and the model parameters are optimized by ISSA. (Reviewer 1, Q1) The separate force, vibration and fusion signal information are brought into the DBN model and the ISSA-DBN model for the prediction of surface roughness, and the results show that the accuracy of the roughness prediction is as follows, respectively DBN: 78.1%, 68.8% and 84.4%, and ISSA-DBN: 93.8%, 87.5% and 100%.

## Introduction

Surface quality is a critical indicator during manufacturing due to its relationship with fatigue strength, deformation and other service performance of the component. However, it requires a lot of effort to realize the evolution of surface quality through theoretical modeling or automatic monitoring^1–4^. Surface roughness is a significant side of the entirely quality of machined parts, which rest with cutting parameters, tool condition, and machine vibrations^5,6^. Surface roughness is important for improving accuracy of assembly, fatigue strength, and resistance of corrosion, therefore, it is identified as a key indicator to estimate the quality of the manufacture components^7–10^. Many scholars are devoted to the monitoring and prediction of workpiece roughness during the machining process, and a large number of techniques are used to construct the relationship between physical phenomena in cutting process and surface roughness.

At present, the estimation of surface roughness can be mainly classified into three methods: theoretical modeling, statistical analysis and artificial intelligence (Reviewer 2, Q1)^11–16^. The theoretical modeling method is used to predict the roughness by analyzing the relationship between the surface roughness generation mechanism and process parameters under different working conditions. The statistical analysis method establishes the statistical regression or classification model of surface roughness through the response surface analysis of the data obtained from the experiment to realize the roughness prediction. The artificial intelligence method is mainly used to realize real-time roughness prediction by establishing one-to-one correspondence between sensor information or process parameters and surface roughness.

Some research has devoted studying on the relationship between milling parameters and roughness directly and exploring the influence of milling parameters on surface roughness. Karkalos et al.^17^ designed experiment for a three factor and three level central composite with the base of Box Behnken Design (BBD). The input parameters including depth of cut, cutting speed and feed rate and surface roughness are selected as output parameters. The quadratic connection among input parameters and surface roughness can be established by RSM. Analysis of variance (ANOVA) was performed to evaluate the arranged formula. Kulisz et al.^18^ measured machinability indices, for example, 3D roughness parameters, chip temperature, chip shape and geometry. Then analysed the machinability of AZ91D magnesium alloy. In general terms, surface roughness parameter is a positive correlation with the feed per tooth *f*_*z*_ and depth of cut *a*_*p*_ parameters. Boga et al.^19^ combined with experiments to think about the influence of different machining parameters, for example tool, feed speed and spindle speed on the surface roughness of high-strength carbon fibre composite plates, and the surface roughness was estimated by using an improved hybrid algorithm. The proposed hybrid ANN-GA algorithm provides a better relationship between surface roughness estimates and measurements.

The transformation of manufacturing from a traditional automated processing mode changed to an intelligent manufacturing model with the speediness development and sustainable improvement of sensor technology. Among them, the multi-sensor fusion system is the only way to realize intelligence. However, a large amount of real-time data generated by multi-sensors is redundant, which increases the complexity of the monitoring model. Therefore, scholars at home and abroad have carried out a lot of research on multi-sensor fusion methods. Maher et al.^20^ used adaptive neuro-fuzzy inference system (ANFIS) modeling to analyze the correlation between cutting parameters, cutting force and surface roughness. In addition, an ANFIS model for accurately predicting the roughness of the end milling surface is established using the cutting force value. Nouhi et al.^21^ combined with 2D surface photography and wavelet method to extract surface textures. Then, the future surface roughness is predicted by extracting the delay parameters, embedded dimensions and pseudo-nearest contiguous values of the resulting surface texture. The prediction error after Ra = 0.4 μm remains basically unchanged in different machining processes. Razfar et al.^22^ comprehensively used the advantages of statistical experimental design technology, experimental measurement, variance analysis, artificial neural network and harmonious search algorithm. Experimental results show that the harmonious search algorithm of fused feedforward neural networks is an efficient and accurate global minimum value approximation method for surface roughness. Misaka et al.^23^ aimed to develop a less data-dependent but effective modeling method. By predicting the surface roughness of the cutting speed, feed speed, cutting depth and three acceleration components of the accelerometer, the Co-Kriging method is compared with the ordinary Kriging method, which confirms that the Co-Kriging method is improved with less model construction data. At the same time, when the measurement data is enough to span the parameter space, the prediction accuracy of the ordinary Kriging method, which only relies on data, is better. (Reviewer 1, Q6) Shah et al.^24^ propose a TCM system that incorporates the Walsh–Hadamard transform for signal processing, DCGAN aims to circumvent the issue of the availability of limited experimental dataset. The results show that the feature selected through Dragonfly algorithms exhibited the least MSE, RMSE, and MAE with a recurrent neural network model, which could help manufacturing companies save money on repairs and replacements, as well as reduce overall production costs by minimizing downtime. Durdy et al.^25^ investigate how kernel approximation functions can be used to better separate data to enhance LOCO-CV applications. They recommend kernelised LOCO-CV as a training paradigm for those looking to measure the extrapolatory power of an algorithm on materials data. Lu et al.^26^ compared the GPR prediction performance of CGI milling experiments with cross-verification, reverse propagation neural network (BPNN) and support vector machine (SVM). Experimental results devoted that the predictive performance of non-cross-verified GPR is similar to that of cross-verified GPR (GPRCV), and both are better than BPNN and support vector machines.

(Reviewer 2, Q1) Although the above studies have achieved good surface roughness prediction results in some certain cases, the fields of the collected cutting signals are not comprehensive, and it is difficult to completely and accurately reflect the surface roughness state of the parts^27–29^. Therefore, the multi-sensor fusion and deep learning network are proposed in this study to improve the accuracy and reliability of surface roughness prediction. Firstly, the vibration and milling force signals are collected during the milling process of die steel P20, and the energy entropy of each component after variational mode decomposition (VMD) is calculated. Secondly, genetic algorithm based mutual information (GA-MI) is taken to cut down the dimension of the original feature data and select the features with high correlation with surface roughness. Since the time series characteristics of the surface roughness data set are not obvious, the deep belief network (DBN) with strong classification and recognition ability is taken to estimate the surface roughness, and the improved sparrow search algorithm (ISSA) is taken to consummate the weight parameters of the DBN model to accelerate the surface roughness prediction accuracy. Furthermore, the Smote algorithm is used to expand the data set to meet the network training requirements with deeper layers.

## Methodology

### Basic structure of deep belief networks

**Figure 1:**
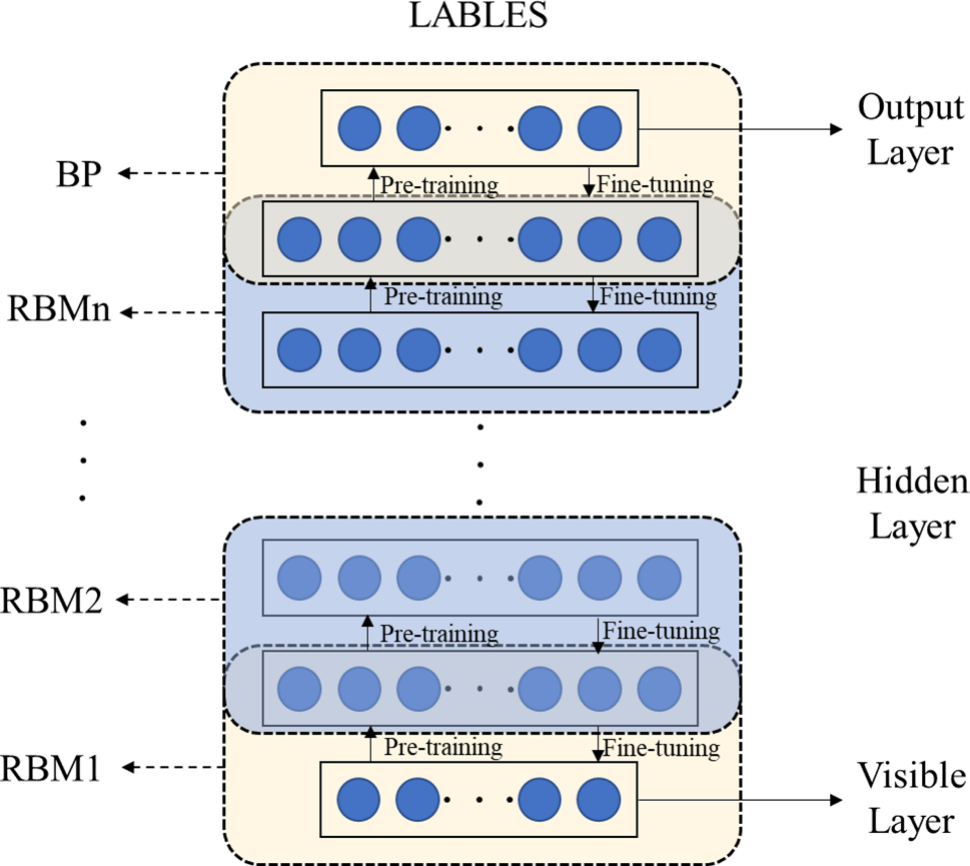
Structure of DBN model.

DBN is a probabilistic generative network consisting of a series of Restricted Boltzmann Machine (RBM) and BP neural networks^30^. As shown in Fig. 1, The DBN network includes a visible layer, n hidden layers and an output layer. The visible layer, that is, the input layer, is located at the base of the structure and input feature sequence. During the learning process, the feature is extracted to traverse multiple hidden layers. In the pre-training process, unsupervised greedy learning algorithms are used to train each RBM layer by layer coming from below, and each layer of network parameters are gradually adjusted to fuse features. The top-down supervision training of the BP neural network is to add tag data to the output layer of DBN during fine-tuning process, propagate the training error to RBM, and fine-tune the parameters of each layer to obtain the global optimal parameters of DBN^31–33^.

(1) Pre-training of RBM.

**Figure 2:**
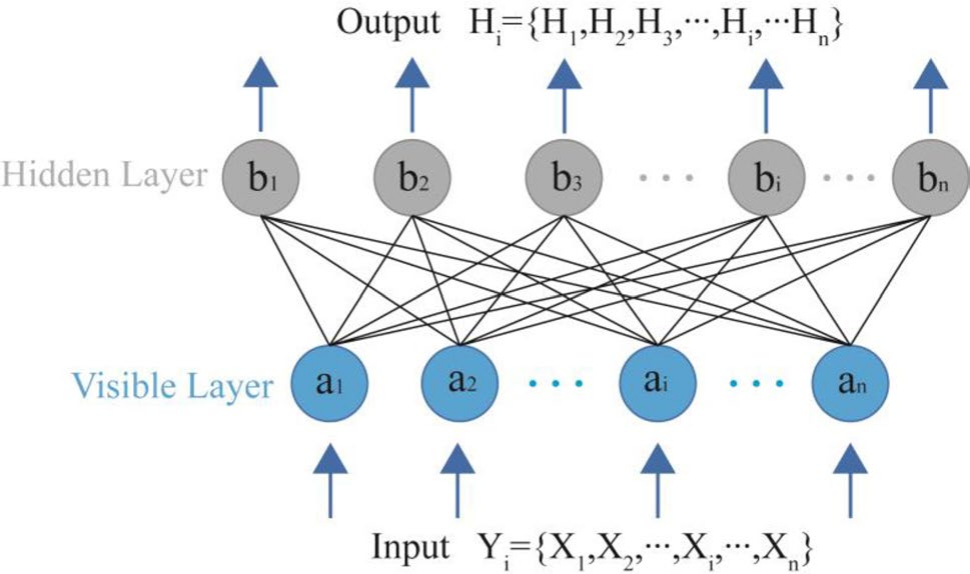
Structure of RBM model.

The RBM includes a visible layer and a hidden layer, and the RBM is discussed in this study using the binary method. As shown in Fig. 2, there is a bidirectional connection in the middle of the two layers, while there is no relation in the middle of the cells of layers. Suppose the number of units in the visible layer is m. The visible layer states are denoted by the vector *v* = {*v*_1_, *v*_2_, …, *v*_*m*_}; simultaneously, the number of units in the hidden layer is n. The hidden layer states are denoted by the vector *h* = {*h*_1_, *h*_2_, …, *h*_*n*_}.

The energy function of RBM is defined as follows:

1 $$E\left(v,h;\theta \right)=-\sum_{j=1}^{m}\sum_{k=1}^{n}{\omega }_{jk}{v}_{j}{h}_{k}-\sum_{j=1}^{m}{a}_{j}{v}_{j}-\sum_{k=1}^{n}{b}_{k}{h}_{k}$$

where *θ* denotes the set of RBM parameters *a*_*j*_, *b*_*k*_, and *ω*_*jk*_, where *a*_*i*_ denotes the unit deviation of the visible layer, *b*_*k*_ denotes the hidden layer unit deviation, and *ω*_*jk*_ denotes the connection weight between the nodes of the input layer and the hidden layer.

Calculate the joint distribution of the layers by the RBM:

2 $$p\left(v,h\right)=\frac{1}{R(\theta )}{e}^{-E(v,h)}$$

3 $$R\left(\theta \right)=\sum_{v,h}{e}^{-E(v,h)}$$

where *R*(*θ*) is the normalization factor and the independent probability distribution of the visible layers is:

4 $$p\left(v\right)=\sum_{h}p\left(v,h\right)=\frac{1}{R(\theta )}\sum_{h}{e}^{-E(v,h)}$$

Since no connection exist in the middle of the nodes, the conditional probability distributions are obtained as follows:

5 $$p\left({h}_{k}=1|v;\theta \right)=\upsigma \left(\sum_{j=1}^{m}{\omega }_{jk}{v}_{j}+{b}_{k}\right)$$

6 $$p\left({v}_{j}=1|h;\theta \right)=\sigma\left (\sum_{k=1}^{n}{\omega }_{jk}{h}_{k}+{b}_{j}\right)$$

where $\sigma (x)=\frac{1}{1+\mathrm{exp}\left(x\right)}$ denotes the activation function from which the probability of hidden layer neurons taking values is calculated from the visible layer and parameters.

The main idea of RBM is to maximize the probability *p*(*v*) values by adjusting the biases *a*_*j*_, *b*_*k*_ and weights *ω*_*jk*_. The set of RBM parameters *θ* = {*a*_*j*_, *b*_*k*_, *ω*_*jk*_} can be get from the sample with the maximum likelihood estimation method with the following gradients for each parameter.

7 $$\frac{\partial lnp(v)}{\partial {\omega }_{jk}}={\langle {v}_{j}{h}_{k}\rangle }_{data}-{\langle {v}_{j}{h}_{k}\rangle }_{model}$$

8 $$\frac{\partial lnp(v)}{\partial {a}_{j}}={\langle {v}_{j}\rangle }_{data}-{\langle {v}_{j}\rangle }_{model}$$

9 $$\frac{\partial lnp(v)}{\partial {b}_{k}}={\langle {h}_{k}\rangle }_{data}-{\langle {h}_{k}\rangle }_{model}$$

where ${\langle \cdot \rangle }_{data}$ denotes the expectation of the current RBM model data distribution and ${\langle  \cdot \rangle }_{model}$ denotes the expectation of the reconstructed RBM model data distribution. The parameter *θ* is updated with the method of contrast scattering.

10 $${{\omega }_{ik}}^{(t+\Delta t)}={{\omega }_{jk}}^{(t)}+\frac{\alpha }{\beta }({\langle {v}_{j}{h}_{k}\rangle }_{data}-{\langle {v}_{j}{h}_{k}\rangle }_{model})$$

11 $${{\omega }_{j}}^{(t+\Delta t)}={{\omega }_{j}}^{(t)}+\frac{\alpha }{\beta }({\langle {v}_{j}\rangle }_{data}-{\langle {v}_{j}\rangle }_{model})$$

12 $${{\omega }_{k}}^{(t+\Delta t)}={{\omega }_{k}}^{(t)}+\frac{\alpha }{\beta }({\langle {h}_{k}\rangle }_{data}-{\langle {h}_{k}\rangle }_{model})$$

where *α* is the learning rate and *β* is the step size.

**Figure 3:**
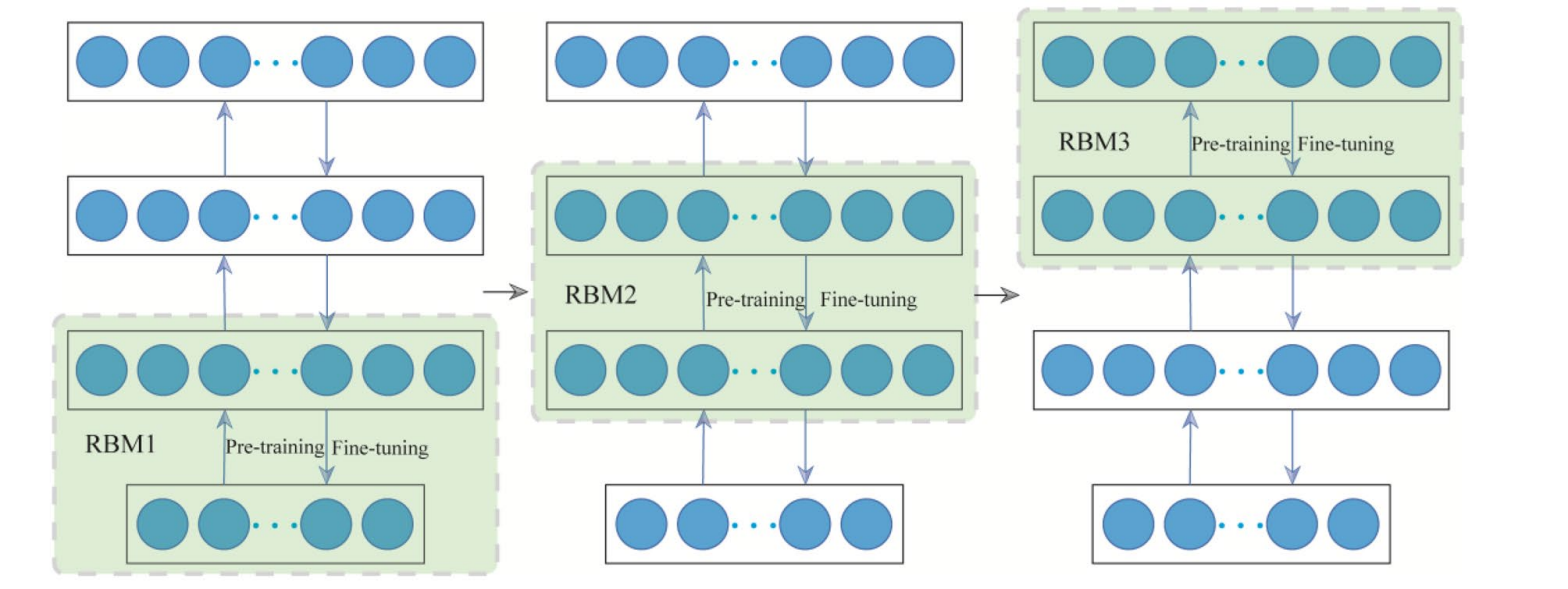
Training process of RBM.

In the back of the completion of the RBM training of the first time, the current hidden layer becomes the visible layer of the next RBM, as shown in Fig. 3. After training each RBM, extracting depth features layer by layer from the original sequence of features.

(2) Fine-tuning of DBN.

For obtaining the global optimization parameters of the DBN model, all layers of RBM network can only make sure that the weights reach the optimal mapping of the feature vector of this layer, rather than the optimal mapping of the feature vector of the entire DBN. Therefore, the error information to all layers of RBM from top to bottom to fine-tune the entire DBN network be propagated by the backpropagation network. With the process of the RBM network, DBN can surmount the shortcomings of the BP network.

### Research on the improved sparrow search algorithm

Sparrow search algorithm (SSA)^34^, is a group intelligent optimization algorithm, which is mainly inspired by foraging and anti-predatory of sparrow. The existing SSA is easy to go on for the optimal solution when solving the problem, resulting in insufficient search range and easy to fall into local convergence, leading to insufficient search accuracy. The improved SSA (ISSA) is proposed to address the problems by using Sin chaos mapping to initialize the population and build up the global search capability. The boundary exploration mechanism is introduced to enhance the boundary search capability and reduce the computing time of the algorithm. In this study, ISSA algorithm is taken to consummate the relevant weight parameters in the DBN model to ameliorate the recognition precise of the model and reduce the interference of uncertainty in the DBN model.

(1) Basic theory of SSA algorithm.

The SSA algorithm is a group intelligence optimization algorithm, which is on account of the biological habits of sparrow foraging and avoiding predators. It mainly simulates the foraging process of sparrows^35^. According to the division of labor, the discoverer is in charge of supplying food and feeding areas for the sparrow population are the two categories divided from the sparrow population, while followers follow the finders at all times, foraging in their vicinity or competing with them for food. Discoverers and followers need to constantly adjust their positions to obtain better positional attributes, but their respective shares in the group are constant. In the foraging process, a certain percentage of individual sparrows are selected for reconnaissance and early warning: alerters. When the alarm value exceeds the limitation of safe, the producer takes the follower to else safe place for food and defines the position as the worst position^36,37^.

The position of each sparrow in the c-dimensional solution space is *X*_*i*_ = (*x*_*1*_, *x*_*2*_, …, *x*_*c*_) and the fitness value *f*_*i*_ = *f*(*x*_*1*_, *x*_*2*_, …, *x*_*c*_). The RMSE in the process of DBN training is used as the fitness function, and the less the fitness the upper the level of sparrow energy reserve. Assuming that there are n virtual sparrows searching for food, the location of the number can be expressed as:

13 $$\mathrm{X}=\left[\begin{array}{cccc}{x}_{\mathrm{1,1}}& {x}_{\mathrm{1,2}}& \cdots & {x}_{1,c}\\ {x}_{\mathrm{2,1}}& {x}_{\mathrm{2,2}}& \cdots & {x}_{2,c}\\ \vdots & \vdots & \vdots & \vdots \\ {x}_{n,1}& {x}_{n,2}& \cdots & {x}_{n,c}\end{array}\right]$$

The fitness values can be expressed as:

14 $${F}_{X}=\left[\begin{array}{c}f([{x}_{\mathrm{1,1}}{x}_{\mathrm{1,2}}\cdots {x}_{1,c}])\\ f([{x}_{\mathrm{2,1}}{x}_{\mathrm{2,2}}\cdots {x}_{2,c}])\\ \cdots \\ f([{x}_{n,1}{x}_{n,2}\cdots {x}_{n,c}])\end{array}\right]$$

where *f* denotes the fitness value of the individual.

The location of the discoverer is updated at each iteration by the following formula:

15 $${X}_{i,j}^{t+1}=\left\{\begin{array}{c}{X}_{i,j}^{t} \cdot \mathrm{exp}\left(-\frac{i}{\alpha  \cdot {iter}_{max}}\right) {R}_{2}<ST\\ {X}_{i,j}^{t}+Q \cdot L {R}_{2}\ge ST\end{array}\right.$$

where *t* is the current iteration and *iter*_*max*_ is the maximum number of iterations. *X*_*i*,*j*_ denotes the position information of the $i$ th sparrow in the jth dimension, *j* = 1,2,…,*c*. *α* ∈ [0, 1] is a random number, *R*_2_ ∈ (0,1] is the alarm value, *ST* ∈ (0.5,1] is the safety value, *Q* is a normally distributed random number, and L is a matrix whose elements are all 1 × *d* matrix with elements of 1. When *R*_2_ < *ST*, it indicates that the foraging area is safe and the producer can search extensively in the area^33^. When *R*_2_ ≥ *ST*, it purposes that the foraging area dangerous, and the sparrow that finds danger alerts the else sparrows, and all sparrows quickly forage in safe area.

During foraging, the followers need to constantly monitor the finders and compete for food as soon as they find better food, and the positions of the followers are renewed in all iterations by the following equation.

16 $${X}_{i,j}^{t+1}=\left\{\begin{array}{c}Q \cdot \mathrm{exp}\left(\frac{{X}_{worst}-{X}_{i,j}^{t}}{{i}^{2}}\right) i>\frac{n}{2}\\ {X}_{P}^{t+1}+\left|{X}_{i,j}^{t}-{X}_{P}^{t+1}\right| \cdot {{\varvec{A}}}^{+} \cdot L i\le \frac{n}{2}\end{array}\right.$$

where *X*_*P*_ is the first-rate place of the producer, *X*_*worst*_ denotes the global worst place in the current iteration, ***A*** denotes the 1, − 1 matrix of 1 × *C*, and ***A***^+^ = ***A***^*T*^(***AA***^*T*^)^−1^. When $i>\frac{n}{2}$, it means that the ith forager with a lower fitness value is in a hungry state, so it must fly elsewhere to obtain more energy.

The number of general prohibitionists accounts for 10 ~ 20% of the number of the entire sparrow population, and their initial positions are randomly generated as follows.

17 $${X}_{i,j}^{t+1}=\left\{\begin{array}{c}{X}_{best}^{t}+\lambda \left|{X}_{i,j}^{t}-{X}_{best}^{t}\right| {f}_{i}\ne {f}_{g}\\ {X}_{i,j}^{t}+K\left(\frac{\left|{X}_{i,j}^{t}-{X}_{worst}^{t}\right|}{\left({f}_{i}-{f}_{w}\right)+\varepsilon }\right) {f}_{i}={f}_{g}\end{array}\right.$$

where *X*_*best*_ devoted the current global optimal place, *λ* devoted the step control parameter and is a random number obeying a standard normal distribution. *K* ∈ [−1, 1] is a random number and ${f}_{i}$ is the current individual fitness value. ${f}_{g}$ is the global first-rate fitness value in the present iteration and ${f}_{w}$ is the global worst fitness value in the current iteration, and a constant ε with a small value is introduced to avoid the denominator being zero. ${X}_{best}$ indicates the best and safest position. *k* is the coefficient of the manipulated step size, characterizing the direction of the sparrow's advance.

(2) ISSA algorithm basic theory.

(a) Sin chaos initialization population.

Sin chaos mapping is chosen to initialize the population in this study, and its one-dimensional self-mapping expression formula is shown as follows.

18 $$\left\{\begin{array}{c}{x}_{m+1}=\mathrm{sin}\frac{2}{{x}_{m}}, m=\mathrm{0,1},\dots ,N\\ -1\le {x}_{m}\le 1,{ x}_{m}\ne 0\end{array}\right.$$

(b) Boundary search mechanism.

Due to the large uncertainty of sparrow behavior, it is easy for the algorithm to perform boundary calculation due to boundary crossing, so the boundary region needs to be explored to avoid the algorithm from falling into local optimum. *a*_*u*_ and *a*_*l*_ are the upper and lower boundary values, respectively, and the SSA boundary calculation formula is:

19 $${X}_{i}^{t+1}=\left\{\begin{array}{ll}{a}_{u}, \quad {X}_{i}^{t+1}\ge {a}_{u}\\ {a}_{l}, \quad {X}_{i}^{t+1}\le {a}_{l}\end{array}\right.$$

Based on this problem, the consequent operator is added to the SSA boundary calculation formula to improve the algorithm's optimization-seeking capability, as follows:

20 $${X}_{i}^{t+1}=\left\{\begin{array}{c}{a}_{u}-m \cdot q, {X}_{i}^{t+1}\ge {a}_{u}\\ {a}_{l}+m \cdot q, {X}_{i}^{t+1}\le {a}_{l}\end{array}\right.$$

where *m* generally takes the value of 0.05 *a*_*u*_ and *q* is a random number belonging to the interval $(\left.0, 1\right]$.

**Figure 4:**
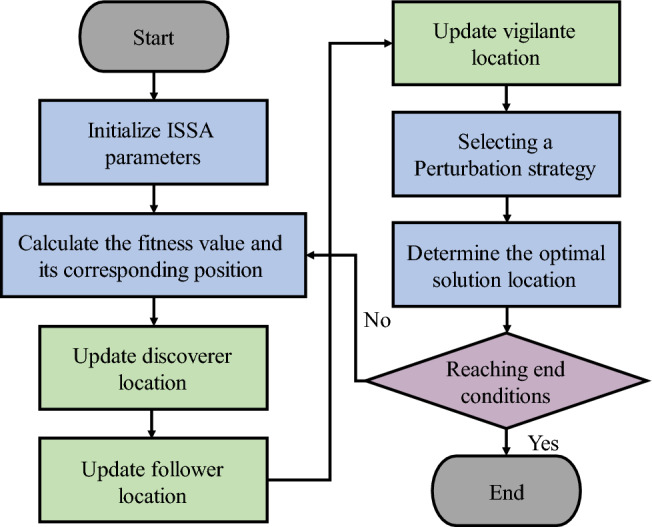
Flow chart of sparrow search algorithm.

The ISSA algorithm flow chart is shown in Fig. 4, with the steps in the next:

Step 1: Initialize the algorithm parameters, such as population size, iterations, proportion of discoverers, proportion of vigilantes, etc.

Step 2: Calculate the fitness values of all individuals to obtain ${f}_{g}$ and ${f}_{w}$ and their corresponding positions.

Step 3: Select some of the sparrows with smaller fitness values as finders and update the finder positions.

Step 4: Remove the discoverer and update the follower position with the remaining sparrows as followers.

Step 5: Select 20% of the sparrows as vigilantes and update the location of the vigilantes.

Step 6: Perturb the current optimal solution by selecting a strategy based on probabilistic *P*_*S*_ to go on a new measure;

Step 7: Determine whether the optimal solution position is updated according to the greedy rule formula;

Step 8: Judge the end condition. If it is the end of the program, output the optimal solution, otherwise repeat steps 2–7.

### Construction of ISSA-DBN model

**Figure 5:**
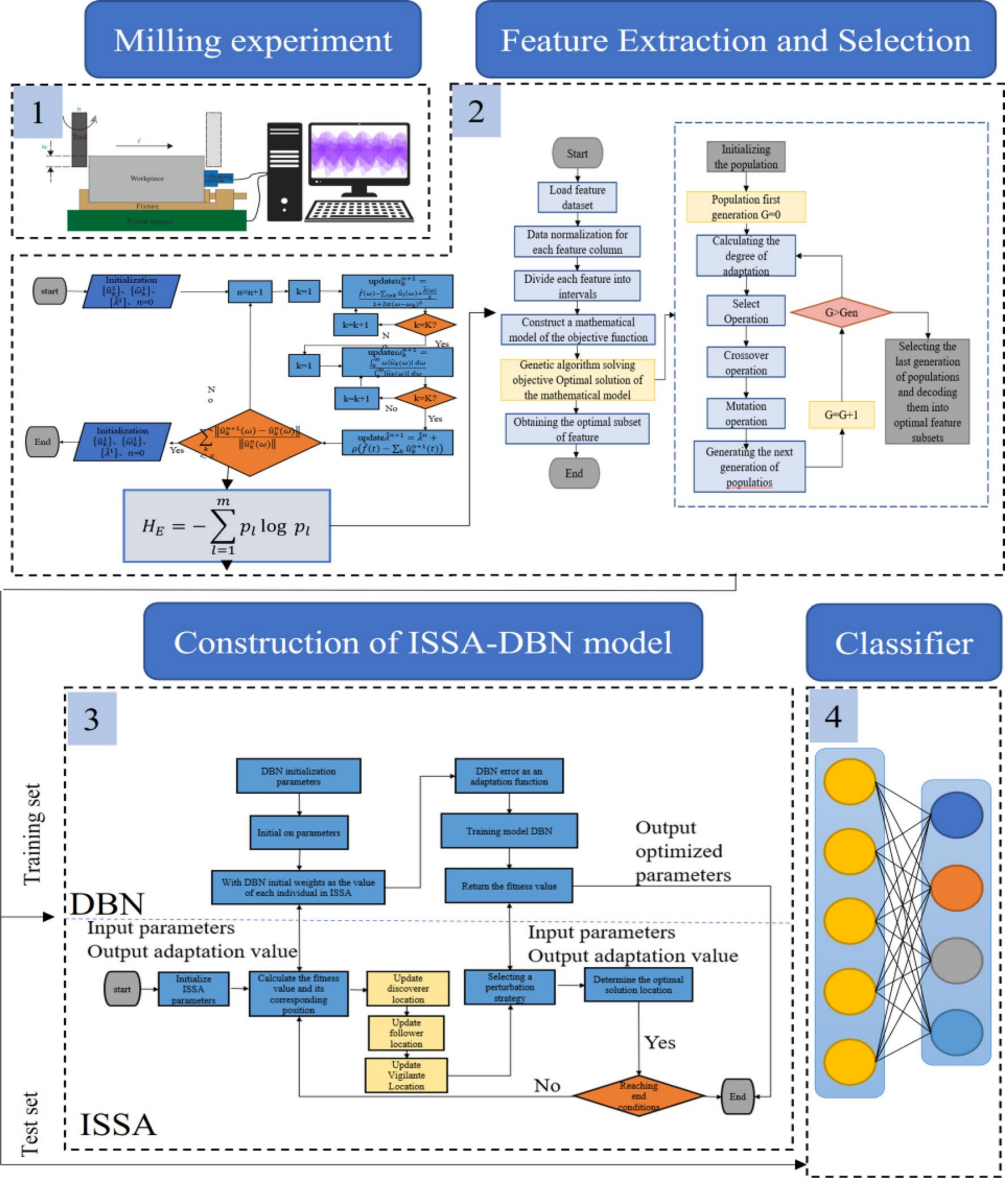
Flow chart of ISSA-DBN architecture.

The optimization process of the ISSA-DBN model is shown in Fig. 5, with the steps as follow:

Step 1: Set the learning rate *γ*, *iter*_*max*_ and other relevant hyperparameters in the DBN model, and the value range of the weight parameter is [−1, 1].

Step 2: Set parameters such as discoverer ratio, follower ratio, and baner ratio of ISSA algorithm.

Step 3: Replace the weight matrix in the DBN model with the values of the location parameters of the individual sparrows in the ISSA algorithm.

Step 4: The initial weights of the DBN model are randomly generated, and the minimized recognition error rate of the DBN model is used as the value of the fitness function of ISSA, which is calculated as follows:

21 $$fitness=1-\frac{sum({y}_{pre}=={y}_{tur})}{N}$$

where *N* is the number of samples, *y*_*pre*_ is the predicted label, *y*_*tur*_ is the true label, and *sum* is the number of predicted and true labels that are the same.

Step 5: Update the ISSA algorithm with the formula in Section "Research on the improved sparrow search algorithm" and assign the optimized weight parameters to the DBN network model, and then obtain the new fitness function values.

Step 6: Sort the fitness function values and select the set of sparrow individual position parameters with the smallest fitness function values, i.e., the optimal weight parameters of the DBN model.

### GA-MI based feature selection method

**Figure 6:**
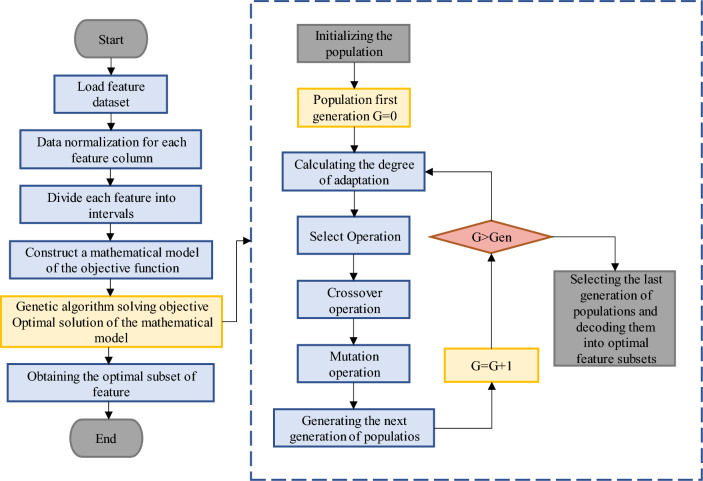
Flow chart of feature selection for GA-MI.

(Reviewer 1, Q4) (Reviewer 2, Q2)When predicting the processing process, the data involved in the operation should be those obtained after removing redundancy and irrelevance that can characterize the changes in the processing progress to ensure high accuracy of model prediction and recognition. Therefore, it is necessary to reduce the dimensionality of the original feature data and select the features with high correlation. The specific process of GA-MI algorithm feature selection is shown in Fig. 6, which mainly consists of two procedures:

1. Genetic algorithm: the genetic algorithm in this study is used to determine the objective function of MI model, which includes initialization, generation, selection, crossover, mutation steps. The last generation of population will be decoded into optimal feature subsets.
2. Mutual Information: the mutual information statistics that measures the degree of dependency or association between two random variables, namely the different signal features generated in the milling process. It quantifies how much information is shared between these variables. MI defines the features at first and then normalize all the features in the same scale. Each features will be divided into different interval and then a model will be constructed based on the objective function. The features and theirs subsets will be evaluated by the fitness values and the features with highest values could be chosen for the inputs of the prediction model.

The mapping relationship between feature values and processing process changes is used as the chromosome fitness value, and the chromosomes of input features are encoded in a binary way, and the optimal combination of features that can accurately predict the processing process is obtained through multiple selection, crossover and variation operations^34,35^.

In this study, the adaptation function is designed based on MI and RMSE, and the adaptation function for each chromosome is calculated as follows:

22 $${F}_{tns}\left({s}_{i}\right)=\alpha *\sum \mathrm{\varnothing }\left(D,R\right)+\gamma *\left(1/\mathrm{RMSE}\right)$$

23 $$\mathrm{RMSE}=\frac{1}{N}{\sum }_{i=1}^{N}{\left({Act}_{i}-{Pred}_{i}\right)}^{2}$$

where: α and γ denote the weight parameters, *N* denotes the number of samples, *Act*_*i*_ denotes the actual value, *Pred*_*i*_ denotes the predicted value, and both α and γ take values in the range [0.1, 1].

## Experimental setup

**Figure 7:**
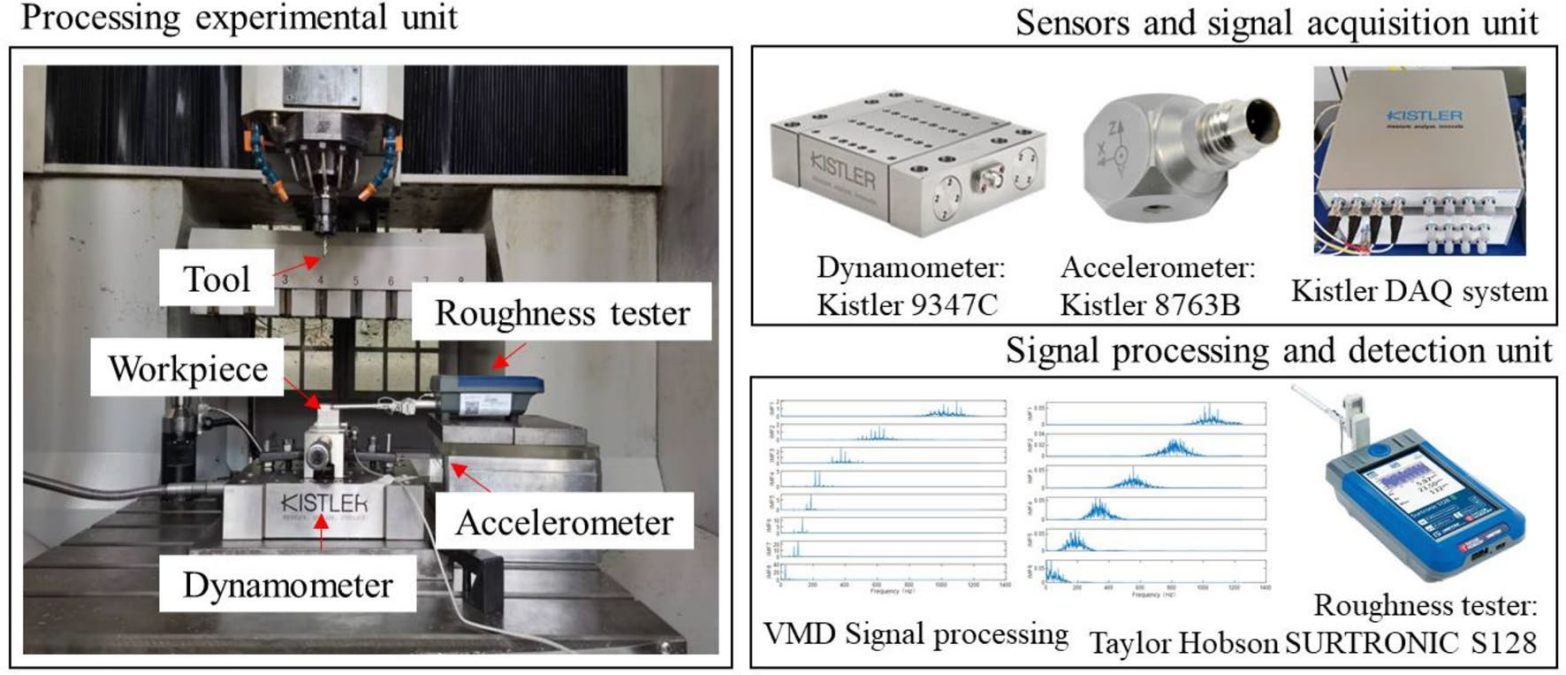
Diagram of experimental setup.

A multi-sensor fusion experiment platform driven by physical signals and processing status data is built on the CarverS600B CNC machine tool, as shown in Fig. 7. It could be divided three parts: processing experiment unit, sensor and signal acquisition unit, and signal processing and detection unit.

1. Processing experimental unit.

In the experimental platform, the dynamometer is fixed on the machine tool table through its matching base, the workpiece is fixed above the dynamometer through the fixture, and the acceleration sensor is directly attached to the side of the workpiece by magnetism. The cutting experiment is carried out by side milling and dry cutting. The workpiece material is P20 die steel with size of 75 mm × 30 mm × 50 mm. The tool is an end mill with diameter of 6 mm by high speed steel. The specific process parameters are planned as follows: the spindle speed *v* ranges from 150 to 200 m/min, the cutting depth *a*_*p*_ ranges from 0.1 to 0.25 mm, and the feed per tooth *f*_*z*_ ranges from 0.025 to 0.05 mm/r.

(2) Sensor and signal acquisition unit.

The milling force and vibration signals are simultaneously collected by the dynamometer Kistler 9347C and accelerometer Kistler 8763B. The nominal sensitivities of dynamometer are Fx: − 8.332 pC/N, Fy: − 4.343 pC/N and Fz: − 8.323 pC/N, while the nominal sensitivities of accelerometer are Ax: 48.43 mv/g, Ay: 53.29 mv/g and Az: 52.53 mv/g.

The multi-channel data acquisition system of DynoWare software are used to receive the signals and the sampling frequency of the acquisition system is set to 2500 Hz for both force and vibration signals.

(3) Signal processing and detection unit.

Statistical and informatics methods are used to analyze and process the physical signal data obtained by the signal acquisition unit, select the signal characteristics that can reflect the dynamic changes of the cutting process, and complete the monitoring of the cutting process based on the neural network model. The detection of surface roughness is accomplished by a portable roughness tester, Taylor Hobson Surtronic S-128.

## Feature processing for prediction of surface roughness

### Feature extraction for surface roughness

In order to provide candidate features containing enough information for feature selection to build an accurate workpiece surface roughness monitoring model, 10 time-domain features and 3 frequency-domain features, (Reviewer 1, Q5) which are listed in Table 1, were extracted from the milling force and vibration signals, respectively, yielding a total of 13 × 5 = 65  features.

**Table 1:** List of signal features.

Time-domain features			Frequency-domain features		
No	Feature	Calculation	No	Feature	Calculation
1	Peak-to-peak	${x}_{p-p}={x}_{max}-{x}_{min}$	11	Frequency centroid	${f}_{c}=\frac{\sum_{i=1}^{N}{f}_{i}{p}_{i}}{\sum_{i=1}^{N}{p}_{i}}$
2	Variance	${x}_{var}=\frac{1}{N}\sum_{i=1}^{N}{(x}_{i}-\overline{x })$			
3	STD	${x}_{std}={\left(\frac{1}{N}{\sum }_{i=1}^{N}({x}_{i}-\overline{x })\right)}^{1/2}$			
4	RMS	${x}_{rms}={(\frac{1}{N}{\sum }_{i=1}^{N}{{x}_{i}}^{2})}^{1/2}$	12	Frequency variance	${v}_{f}=\frac{\sum_{i-1}^{N}{({f}_{i}-{f}_{c})}^{2}{p}_{i}}{\sum_{i=1}^{N}{p}_{i}}$
5	Skewness	${x}_{sk}=\frac{1}{n-1}\frac{\sum_{i=1}^{N}{({x}_{i}-\overline{x })}^{3}}{{{x}_{std}}^{3}}$			
6	Kurtosis	${x}_{sk}=\frac{1}{n-1}\frac{\sum_{i=1}^{N}{({x}_{i}-\overline{x })}^{4}}{{{x}_{std}}^{4}}$			
7	Crestor factor	$\mathrm{S}=\frac{{x}_{rms}}{\frac{1}{N}\sum_{i=1}^{N}\left|{x}_{i}\right|}$	13	Mean square frequency	${ms}_{f}=\frac{\sum_{i=1}^{N}{{f}_{i}}^{2}{p}_{i}}{\sum_{i=1}^{N}{p}_{i}}$
8	Peak factor	$\mathrm{C}=\frac{{x}_{p-p}}{{x}_{rms}}$			
9	Pulse factor	$\mathrm{S}=\frac{{x}_{p-p}}{\frac{1}{N}\sum_{i=1}^{N}\left|{x}_{i}\right|}$			
10	Clearance factor	$\mathrm{L}=\frac{{x}_{p-p}}{{(\frac{1}{N}\sum_{i-1}^{N}{\left|{x}_{i}\right|}^\frac{1}{2})}^{2}}$			

The cutting signals were decomposed using variational modal decomposition, and the energy entropy of each modal component obtained from the decomposition was able to characterize the roughness variation from different scales. The energy entropy of the *l*^th^ IMF was calculated as follows.

24 $${H}_{E}=-\sum_{l=1}^{m}{p}_{l}\mathrm{log} {p}_{l}$$

25 $${p}_{l}=\frac{{E}_{l}}{{E}_{sum}}$$

where ${E}_{l}$ is the energy of the *l*^th^ IMF component and ${E}_{sum}$ is the energy sum of all components.

**Figure 8:**
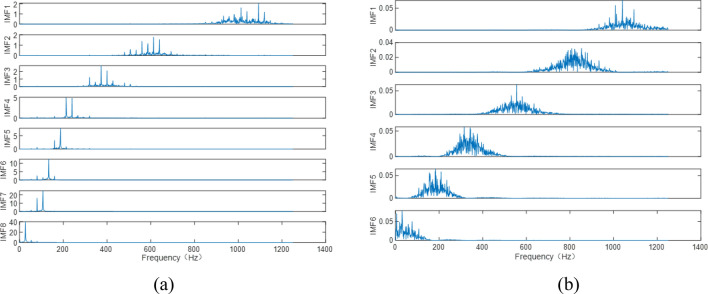
Frequency domain diagram of IMF component: (**a**) variational modal decomposition of the X-direction milling force signal; (**b**) variational modal decomposition of the X-direction vibration signal.

The optimal number of modes for the variable modal decomposition of milling force and vibration signals are 8 and 6. The frequency centers of each IMF component are their respective center frequencies, and there is no frequency mixing, which indicates that the adopted method works well, as show in Fig. 8. The milling force signal is decomposed into 8 IMF components (note that the 8 IMF energy entropy features of milling force are numbered 14–21), and the vibration signal is decomposed into 6 IMF components (note that the 6 IMF energy entropy features of vibration are numbered 14–19). In the time–frequency domain, 36 features are obtained.

There are a total of 101 features for workpiece surface roughness prediction (the meaning of features numbered 1–21 (or 1–19) , the feature order of IMF energy entropy, the milling force features of X, Y and Z direction, and the vibration signals of X and Y direction are arranged in this order. The specific distribution of the 101 extracted features is as follows: the features of the X-direction milling force signal are recorded as 1–21, the features of the Y-direction milling force signal are recorded as 22–42, the features of the Z-direction milling force signal are recorded as 43–63, the features of the X-direction vibration signal are recorded as 64–82, and the features of the Y-direction vibration signal are recorded as 83–101).

The X-direction milling force signal is used as an example to observe the change of each feature extracted by the roughness as the experimental sequence number increases.

**Figure 9:**
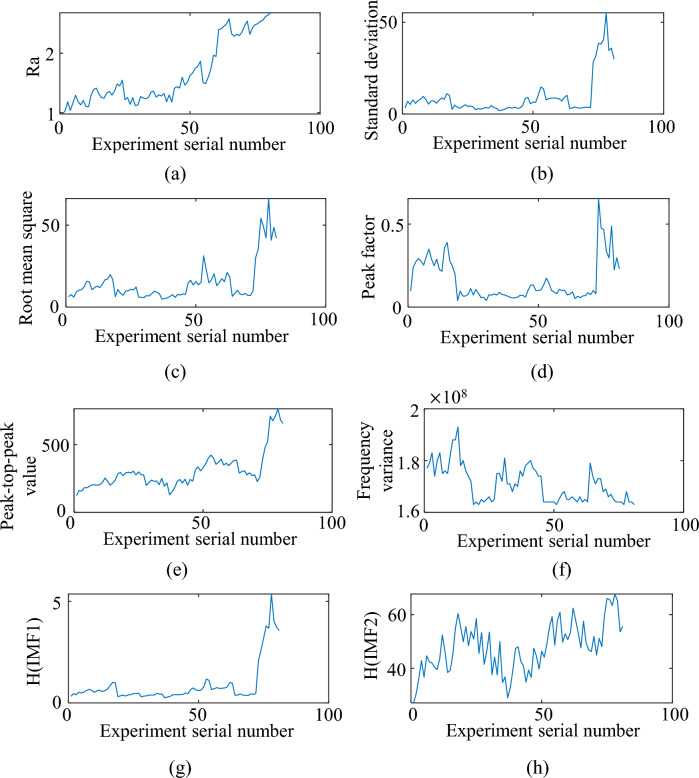
Variation of surface roughness and its monitoring characteristics: (**a**) Surface roughness; (**b**) Standard deviation; (**c**) Root mean square; (**d**) Peak factor; (**e**) Peak-to-peak value; (**f**) Frequency variance; (**g**) IMF1; (**h**) IMF2.

Figure 9 shows the performance of some features extracted from the surface roughness of the workpiece. It can be seen that the trend of the eigenvalues of standard deviation, RMS, peak-to-peak, peak factor, and IMF2 is the same as the trend of Ra values, all of which grow slowly and then sharply. The change trend of feature values such as frequency variance is opposite to the change trend of Ra, showing a gradual decrease, and although the change of feature values is different, there is a significant difference in the feature values as the Ra value increases, so that the above extracted features can be used to discriminate the change of surface roughness state.

### Determination of optimal feature combinations

**Figure 10:**
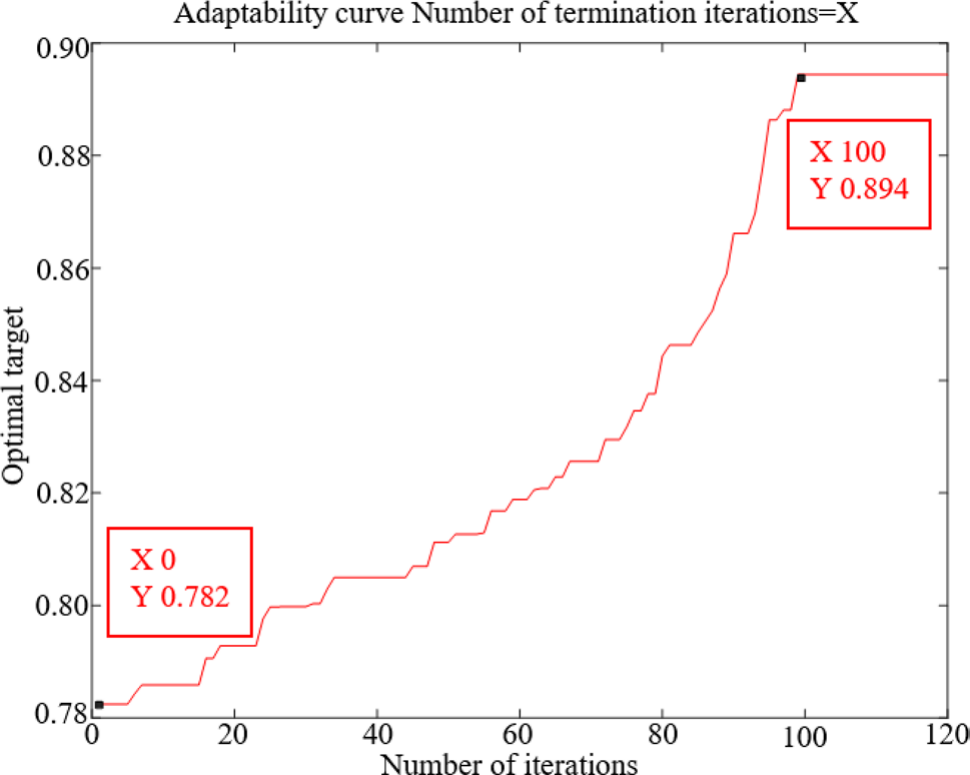
Trend of GA-MI fitness function values.

By reviewing the relevant literature^36,37^, the process parameters for GA-MI based feature selection were set as shown in Table 2. From Fig. 10, it can be known that the value of the fitness function reaches the maximum when the number of iterations reaches 100, after which the value of the fitness function remains stable as the number of iterations future rise, so the number of iterations is set to 100.

**Table 2:** Parameter setting of GA-MI based feature selection.

Parameters	Parameter values
Variance probability	0.1
Crossover probability	0.8
Learning Rate	0.1
*α*	0.2
*γ*	0.7

By using the GA-MI method, the fitness values under the results of different combinations of the number of features were calculated for 101 features from the signal fusion, 63 features from the single milling force and 38 features from the single vibration signal extracted from the roughness orthogonal experimental data set, respectively, and the calculated results are shown in Tables 3, 4 and 5.

**Table 3:** Preferred results of different combinations of feature numbers from signal fusion.

Value of fitness function	Number of features	Specific feature combinations
0.852	5	3, 7, 20, 78, 93
0.873	9	2, 3, 12, 19,23, 29, 55, 77, 85
0.934	12	2, 4, 7, 19, 28, 42, 56, 62, 73, 81, 94, 101
0.958	16	1, 3, 5, 10, 18, 27, 38, 44, 52, 59, 64, 75, 83, 88, 94, 98, 100
0.931	20	1, 2, 4, 5, 12, 17, 21, 24, 30, 33, 42, 52, 59, 64, 69, 72, 77, 85, 97, 101
0.916	26	1, 3, 4, 5, 10, 16, 18, 22, 34, 39, 43, 45,…, 61, 67, 69, 71, 75, 88, 92, 95
0.885	32	1, 2, 4, 5, 10, 12,…, 49, 52, 56, 59, 62, 65, 72, 75, 78, 84, 91, 98,…, 100
0.841	46	1, 2, 3, 5, 10, 12,…, 57, 61, 67, 73, 75, 78, 82, 85, 86, 89, 92, 95, 99, 100
0.801	60	1, 2, 4, 5, 9, 10, 14, 16,…, 47, 53, 54, 56, 58, 62, 64, 65, 68, 71, 73, 74,…, 101
0.732	78	1, 2, 5, 6, 7,…, 23, 24, 25, 27, 29, 31,…, 62, 64, 65, 68, 69, 71, 73,…,92, 98, 100

**Table 4:** Preferred results for different combinations of features from single milling force feature.

Value of fitness function	Number of features	Specific feature combinations
0.862	5	4, 16, 24, 46, 61
0.939	7	1, 4, 12, 25, 34, 46, 62
0.918	10	1, 3, 4, 10, 24, 32, 45, 49, 53, 59
0.829	16	1, 2, 3, 4, 10, 16, 24, 25, 34, 35, 42, 45, 49, 53, 58, 59
0.885	22	1, 2, 4, 5, 10, 14, 16, 24, 25, 27, 31, 34, 38, 42, 45, 47, 49, 51, 53, 57, 59, 63
0.856	30	1, 2, 3, 4, 5, 6, 10, 12, 14, 16, 18,…, 34, 35, 36, 39, 41, 42, 44, 45, 52, 53, 61, 63

**Table 5:** Preferred results for different combinations of the number of features from single vibration feature.

Value of fitness function	Number of features	Specific feature combinations
0.843	5	64, 68, 73, 81, 95
0.892	7	68, 69, 73, 85, 94, 95, 100
0.885	12	64, 68, 69, 72, 73, 78, 84, 85, 92, 95, 100, 101
0.847	18	64, 65, 68, 71, 72, 73, 75, 81, 82, 85, 86, 88, 89, 91, 94, 95, 99, 101
0.820	20	64, 67, 68, 70, 71, 72, 74, 78, 81, 82, 85, 86, 88, 89, 91, 92, 95, 99, 100, 101
0.794	26	64, 65, 68, 69, 71, 73, 74, 75, 77,…, 87, 89, 91, 92, 93, 94, 95, 97, 99, 100, 101

From Tables 3, 4 and 5, the highest fitness values are found for the number of features of 16, 10 and 7, respectively, so the optimal number of feature combinations for the fusion signal, single milling force signal and single vibration signal are 16, 10 and 7, respectively.

The optimal number of feature combinations for single and multiple signals can be known in Tables 3, 4 and 5 as well. This section focuses on the results of the feature combination preferences of GA-MI when the number of features selected under the fusion signal. According to the highest value of fitness function, the number of features in single milling force signal and single vibration signal are 16, 10 and 7, respectively, as shown in Table 6.

**Table 6:** Sensitive Feature Values Preferred by GA-MI Algorithm.

Feature Serial Number	Fusion signal characteristics	Single milling force characteristics	Single vibration characteristics
Feature 1	X-direction milling force peak-to-peak	X-direction milling force peak-to-peak	Vibration variance in X-direction
Feature 2	Variance of milling force in X-direction	X-direction milling force margin factor	X-directional vibration cliff
Feature 3	Frequency variance of milling force in X direction	X-direction milling force wavelet packet node 1 energy share	X-directional vibration wavelet packet node 1 energy share
$\vdots$	$\vdots$	$\vdots$	$\vdots$
Feature 6	Standard deviation of milling force in Y direction	Z-direction milling force peak factor	Vibration frequency variance in Y direction
Feature 7	Y-direction milling force peak factor	Z-direction milling force margin factor	Y-directional vibration wavelet packet node 4 energy share
$\vdots$	$\vdots$	$\vdots$	
Feature 10	Z-direction milling force wavelet packet node 1 energy share	Z-direction milling force wavelet packet node 4 energy share	
$\vdots$	$\vdots$		
Feature 16	Energy share of wavelet packet node 2 in Y-direction vibration		

## Analysis of surface roughness prediction results

### Expansion of the surface roughness dataset

**Figure 11:**
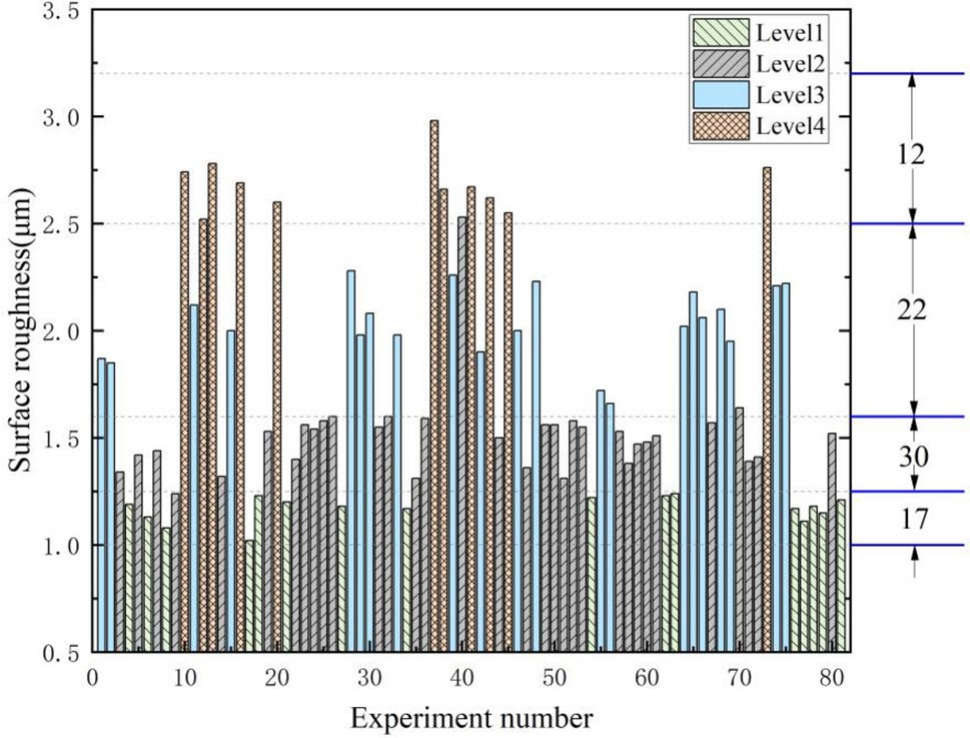
Data distribution of surface roughness.

In the practical production process, there are certain processing requirements for the surface roughness of the parts. However, the interval of Ra values specified in the current national standard is too large, which is not quite applicable to some products with high processing accuracy requirements, so this study combines Ra values and Ra series supplementary values, and divides the Ra values (ranging from 1 to 3.2 μm) in this study into four intervals, naming them as grades 1–4 respectively, and each grade is divided as shown in Fig. 11, and uses ISSA- DBN model was used to identify the roughness classes. Using the model proposed in this study, it is easy to identify whether the product surface roughness is within the desired roughness class, enabling efficient production of parts that meet the machining accuracy and reducing the time cost of multiple downtimes to measure the surface roughness. All data samples of surface roughness were divided according to four classes, and 17, 30, 22 and 12 data samples were obtained for each class.

**Figure 12:**
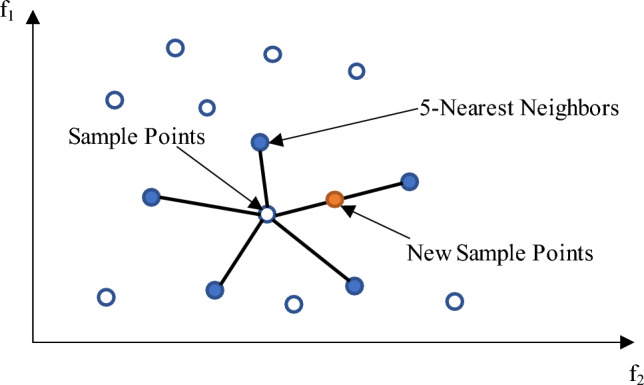
Schematic diagram of SMOTE expansion sample.

The dataset of each level is expanded due to the small number of samples in each level, which may not meet the training needs of networks with deeper layers. Traditional random oversampling uses replication of samples to increase the number of samples, and this method is prone to overfitting because no new samples are generated. The SMOTE algorithm generates new samples by artificially adding new data points to the data set based on the distribution of the original samples, which increases the size of the migratable samples to some extent. The process of generating new sample points for the SMOTE algorithm is shown in Fig. 12.

For each sample point x in the original sample, the distance from it to all sample points in the pattern set S is computed using Euclidean distance to obtain its *k*-nearest neighbor (*k*-nearest neighbor), and a number of samples are wildly chosen from its *k*-nearest neighbors. For the selected *i*th nearest neighbor *x*_*i*_, the relationship equation with the original sample point *x* and the new sample point, is shown as follows.

26 $${x}_{new}=x+rand(\mathrm{0,1})\left|x-{x}_{i}\right|$$

Table 7 shows the comparison of the surface roughness feature dataset before and after the expansion. The training dataset is expanded by about 3.2 times, and since the test set cannot be expanded, the test dataset before and after the expansion remains unchanged.

**Table 7:** Part surface roughness data set.

Grade	Ra ($\upmu \mathrm{m}$)	Pre-expansion dataset		Expanded dataset		Test data set
		Dataset	Training data set classification	Dataset	Training data set classification	
1	1–1.25	81	10	262	65	32
2	1.25–1.6		14		62	
3	1.6–2.5		13		62	
4	2.5–3.2		12		66	

**Figure 13:**
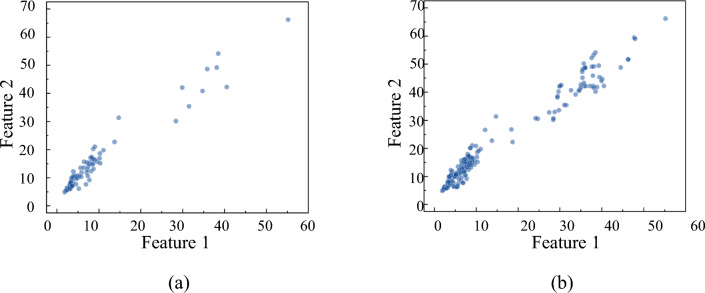
Comparison of training data set before and after expansion: (**a**) training dataset before expansion; (**b**) training dataset after expansion.

Figure 13 shows the training dataset before and after the expansion, and it can be seen from the figure that the expanded dataset has the same trend and is more dense as before the expansion.

### Model parameter setting

The surface roughness features selected in Sect. 4.3 are input into the DBN and ISSA-DBN models to identify the surface roughness classes, respectively. Since the recognition is a classification problem, a SoftMax classifier is added to the output layer, and in this study, four implicit layers are selected in the DBN model with the number of nodes^10,10^. ISSA optimizes the weight parameters of DBN with the recognition error rate as the fitness function. The specific DBN and ISSA specific related parameters are shown in Table 8, where the parameters of SSA are the same as those of ISSA.

**Table 8:** Algorithm parameter settings.

Algorithm	Parameters	Parameter values
DBN	Number of iterations	100
	Learning Rate	0.1
	Loss function	CrossEntropy
ISSA	Number of populations	50
	Number of iterations	100
	Discoverer	20%
	Vigilantes	10%
	Safety Threshold	0.8

### Surface roughness grade identification analysis

#### Analysis of surface roughness grade recognition results of different signal features

(a) Analysis of the results of grade recognition of surface roughness for a single milling force signal feature.

**Figure 14:**
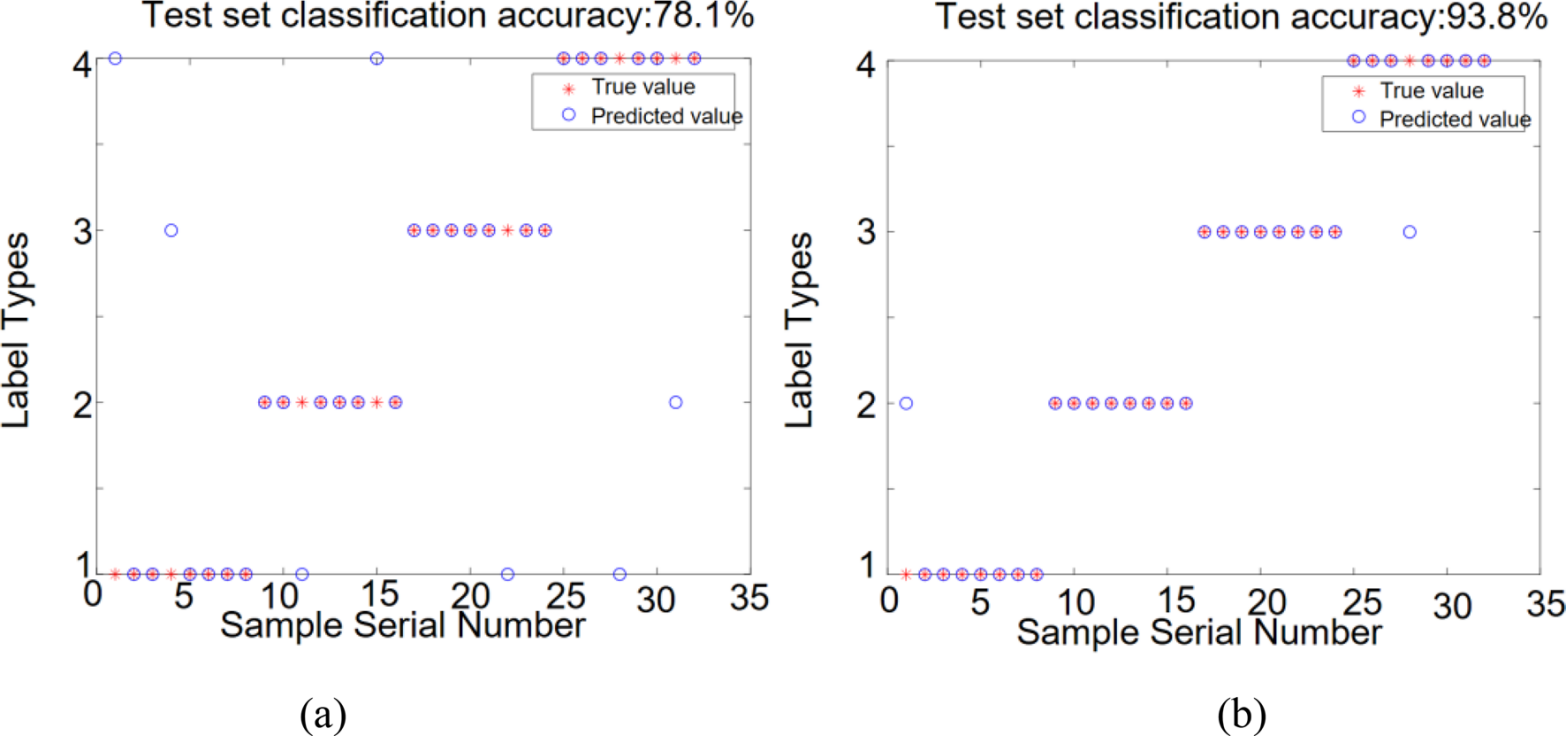
Identification results for different model test sets with a single milling force signal: (**a**) DBN model; (**b**) ISSA-DBN model.

The number of features extracted from a single milling force signal is 10. The expanded feature set is input into the DBN and ISSA-DBN models, and the recognition results are shown in Fig. 14. The horizontal coordinate represents the number of sample serial and the vertical coordinate represents the roughness class category. The true label value is indicated by a red asterisk and the predicted label value is indicated by a blue circle; When the true label value is the same as the predicted label value, the two marks overlap.

**Figure 15:**
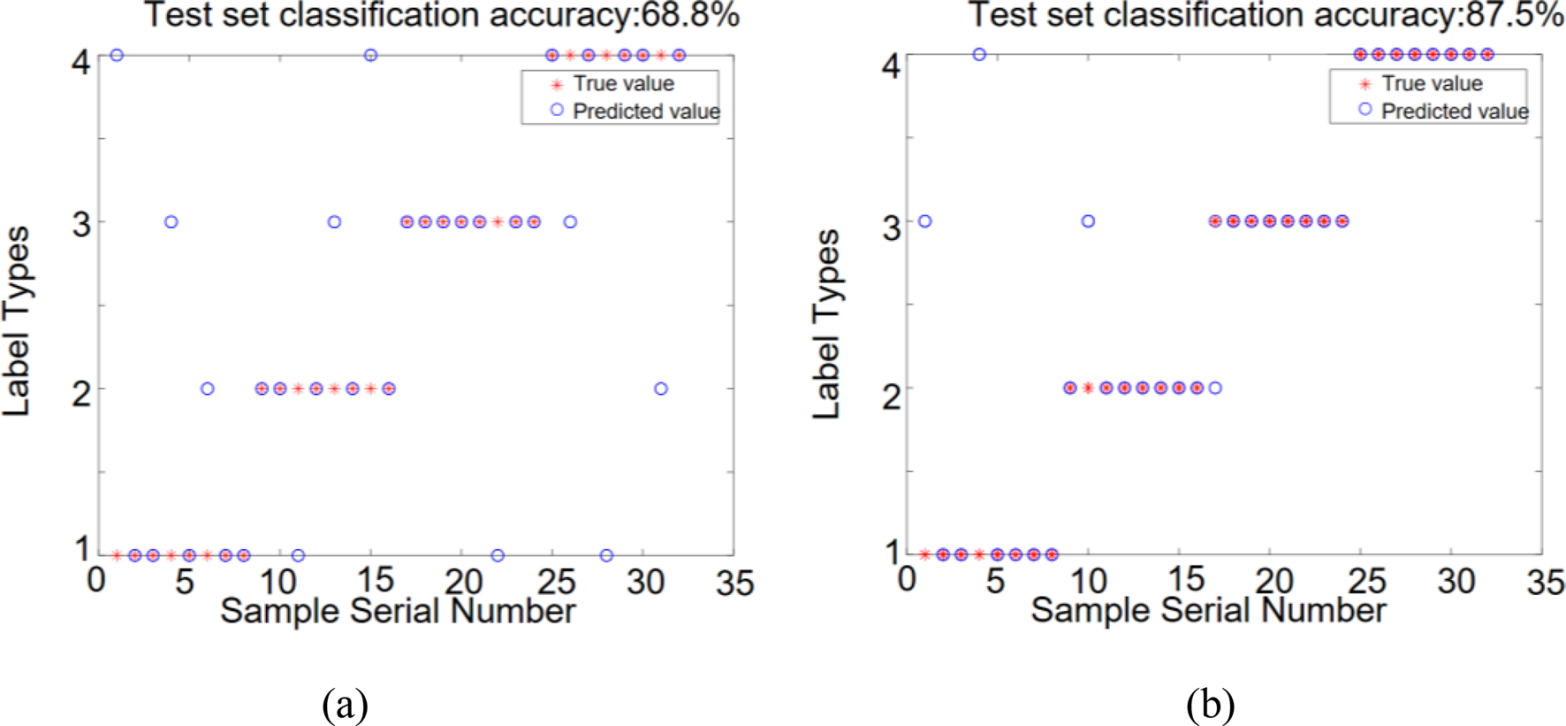
Identification results of different model test sets with a single vibration signal: (**a**) DBN model; (**b**) ISSA-DBN model.

The number of features extracted from a single vibration signal is 7. The expanded feature set is input into the DBN and ISSA-DBN models, and the recognition results are shown in Fig. 15.

**Figure 16:**
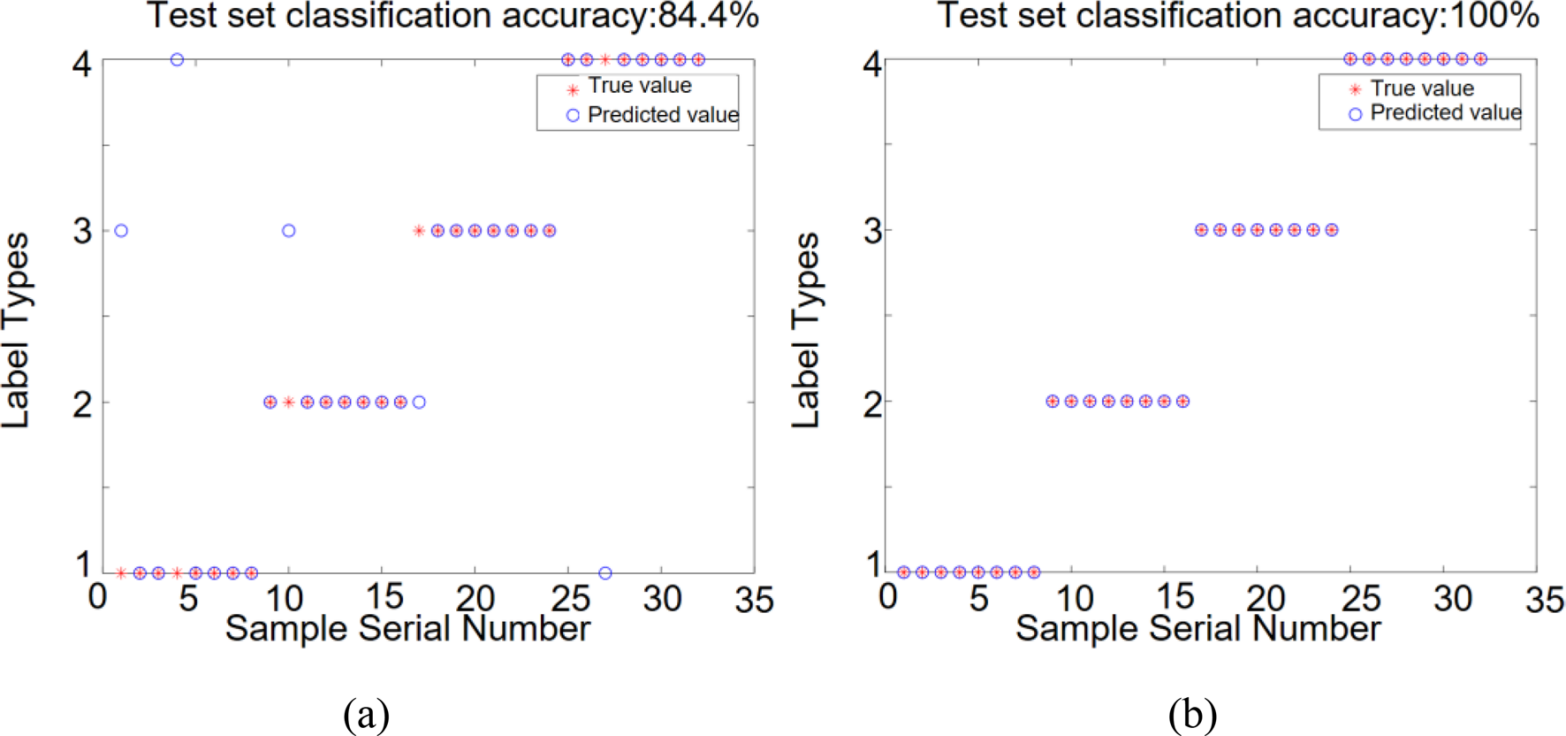
Identification results of different model test sets with force and vibration signal fusion: (**a**) DBN model; (**b**) ISSA-DBN model.

The number of features extracted by fusing the milling force and vibration signals is 16. The expanded feature set is input into the DBN and ISSA-DBN models, and the recognition results are shown in Fig. 16.

From Figs. 14, 15 and 16, it can be obtained that (a) The recognition rate of DBN model relative to ISSA-DBN model on the test set is reduced by 16.7% on average, indicating that the ISSA algorithm has a strong weight parameter optimization capability; (b) The features of fusion signals contain more information related to surface roughness level relative to single signal features, and have higher recognition accuracy; (c) Milling force signal features have higher recognition accuracy than vibration signal features.

(2) Analysis of surface roughness grade recognition results of sample data sets under different models before and after expansion.

The SMOTE algorithm was used to expand the sample datasets for single milling force, single vibration, and fused features after feature selection, respectively. The sample datasets before and after the expansion were used as input to the DBN model and ISSA-DBN model, respectively, for surface roughness identification, and the identification results are shown in Table 9.

**Table 9:** Roughness recognition accuracy of each sample data set under different models before and after expansion.

	DBN (%)		ISSA-DBN (%)		Improved after expansion (%)	
	Before expansion	After expansion	Before expansion	After expansion	DBN	ISSA-DBN
Single milling force	62.5	78.1	75.0	93.8	15.6	18.8
Single vibration	56.3	68.8	68.8	87.5	12.5	18.7
Signal fusion	65.6	84.4	82.3	100.0	18.8	17.7

(Reviewer 2, Q3) As shown in Table 9, the recognition accuracy of the DBN model with single milling force, single vibration, and signal fusion after expansion is 15.6%, 12.5%, and 18.8% higher than that of the model before expansion, respectively. The average improvement is 15.6%. The recognition accuracy of the ISSA-DBN model with single milling force, single vibration, and signal fusion after expansion is 18.8%, 18.7%, and 17.7% higher than that of the model before expansion, respectively. The average improvement is 18.4%. The recognition results show that the recognition accuracy of the expanded dataset is higher than that of the original dataset under different models and different features, and thus, the expanded dataset can be more effective in training a high-precision surface roughness recognition model.

(3) Analysis of the grade recognition results of surface roughness before and after feature selection.

**Figure 17:**
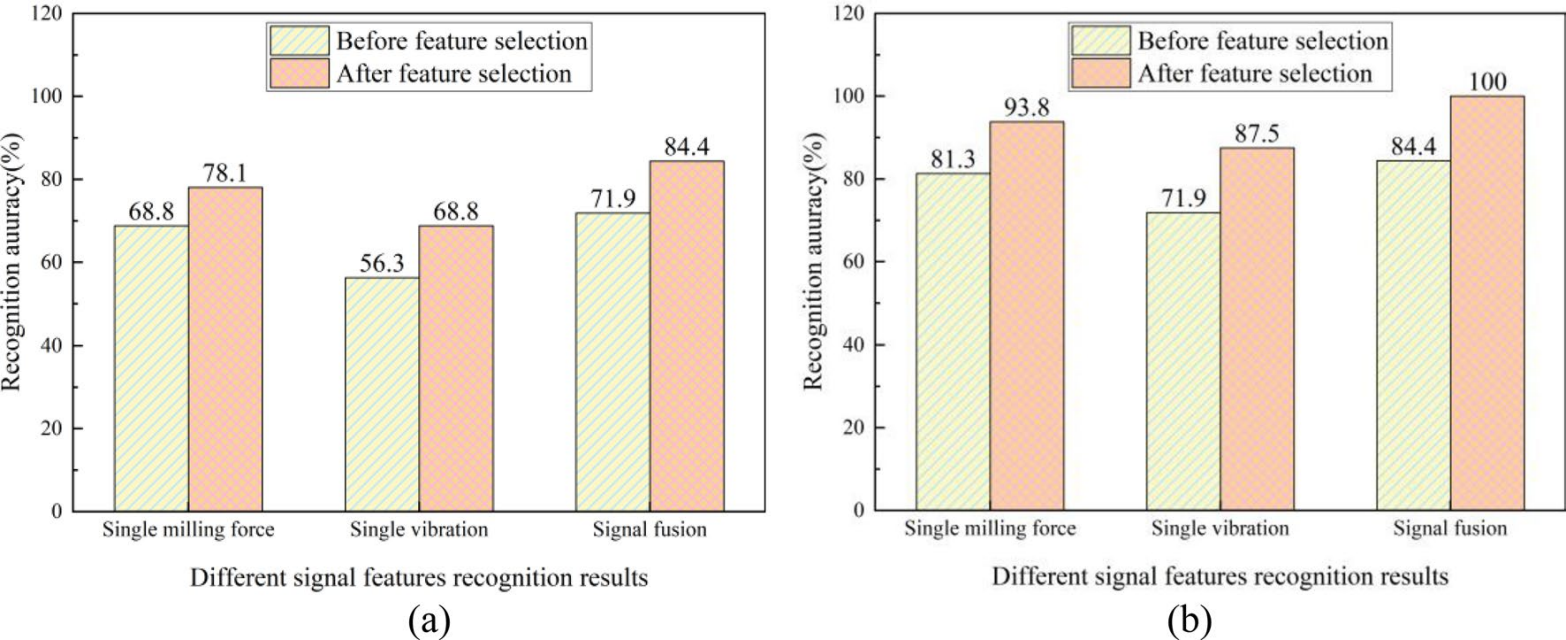
Recognition results of different signal features before and after feature selection: (**a**) DBN model; (**b**) ISSA-DBN.

The expanded datasets of single milling force feature, single vibration feature and fused feature before feature selection and the expanded datasets of single milling force feature, single vibration feature and fused feature after feature selection were used as inputs to ISSA-DBN and the model, respectively, for roughness class recognition, and the recognition accuracy is shown in Fig. 17.

The recognition accuracy of both the DBN model and the ISSA-DBN model after feature selection is higher than that before feature selection, (Reviewer 1, Q8) both for single features (single force or single vibration signal) and fused features (combined force and vibration signals). The recognition accuracy of the DBN model after feature selection is improved by 11.4% on average, and that of the ISSA-DBN model is improved by 14.6% on average. By selecting a subset of features with high correlation and low redundancy, the computational complexity of the prediction model is effectively reduced, which fully proves the necessity of feature selection.

## Conclusions

In this study, roughness experiments were designed to establish a classification model using the response surface method to analyze the response relationship between roughness and cutting parameters. The conclusions of this work can be drawn as follows:

(Reviewer 2, Q4) (1) The milling force and vibration signals are preprocessed to extract effective processing information and remove outliers, and then the processed signals are decomposed using variational modal decomposition to extract 10 time-domain features and 3 frequency-domain features in each IMF of different force and vibration signals.

(2) The GA-MI method is used to select the features with high correlations with surface roughness among dozens of extracted features. The optimal subset of features for the single milling force signal, the single vibration signal, and the fusion signal are selected by the value of fitness function.

(3) The SMOTE algorithm is applied to expand the surface roughness datasets for single milling force, single vibration, and fused features after feature selection. The datasets before and after the expansion are used as the input of DBN model and ISSA-DBN model, respectively, for the comparison of surface roughness prediction.

(4) The prediction accuracy of proposed ISSA-DBN model with optimal feature subsets and data expansion is higher than DBN with optimal feature subsets and data expansion model by 15.6%. The model with optimal feature subsets improves the prediction accuracy by 6.2%, while the model with data expansion improves the prediction accuracy by 17.7%.

### Supplementary Information

Supplementary Information.

## Data Availability

The datasets used and/or analysed during the current study available from the corresponding author on reasonable request. All data generated or analysed during this study are included in this published article and its supplementary information files.
