# Supplementary material for: Prediction of surface roughness based on fused features and ISSA-DBN in milling of die steel P20
Source: Sci Rep. 2023 Sep 24;13:15951. doi: 10.1038/s41598-023-42968-4 (PMC10518346; doi:10.1038/s41598-023-42968-4)
Supplement: Supplementary file 1 — Supplementary Information. [file 41598_2023_42968_MOESM1_ESM.docx]

Appendices

List of experimental parameters

| No. | v/  m/min | ap/  mm | fz/  mm/z | | Ra |
| --- | --- | --- | --- | --- | --- |
|  |  |  |  |  | μm |
| 1 | 150 | 0.1 | 0.025 | 1.87 | |
| 2 | 150 | 0.1 | 0.0325 | 1.85 | |
| 3 | 150 | 0.1 | 0.04 | 1.74 | |
| 4 | 160 | 0.2 | 0.0325 | 1.48 | |
| 5 | 160 | 0.25 | 0.05 | 1.62 | |
| 6 | 180 | 0.15 | 0.05 | 1.41 | |
| 7 | 180 | 0.25 | 0.025 | 1.64 | |
| 8 | 200 | 0.15 | 0.04 | 1.39 | |
| 9 | 200 | 0.2 | 0.025 | 1.28 | |
| 10 | 150 | 0.15 | 0.025 | 2.34 | |
| 11 | 150 | 0.15 | 0.0325 | 2.12 | |
| 12 | 150 | 0.2 | 0.04 | 2.32 | |
| 13 | 160 | 0.1 | 0.025 | 2.37 | |
| 14 | 160 | 0.1 | 0.05 | 1.62 | |
| 15 | 180 | 0.1 | 0.0325 | 2.00 | |
| 16 | 180 | 0.2 | 0.025 | 2.69 | |
| 17 | 200 | 0.25 | 0.04 | 0.93 | |
| 18 | 200 | 0.25 | 0.05 | 1.23 | |
| 19 | 150 | 0.2 | 0.04 | 1.63 | |
| 20 | 150 | 0.25 | 0.025 | 2.30 | |
| 21 | 150 | 0.25 | 0.0325 | 1.49 | |
| 22 | 160 | 0.15 | 0.025 | 1.70 | |
| 23 | 160 | 0.2 | 0.0325 | 1.56 | |
| 24 | 180 | 0.1 | 0.04 | 1.73 | |
| 25 | 180 | 0.15 | 0.05 | 1.58 | |
| 26 | 200 | 0.1 | 0.025 | 1.70 | |
| 27 | 200 | 0.1 | 0.05 | 1.45 | |
| 28 | 150 | 0.2 | 0.025 | 2.28 | |
| 29 | 150 | 0.25 | 0.0325 | 1.98 | |
| 30 | 150 | 0.25 | 0.05 | 2.08 | |
| 31 | 160 | 0.15 | 0.025 | 1.55 | |
| 32 | 160 | 0.15 | 0.04 | 1.60 | |
| 33 | 180 | 0.1 | 0.0325 | 1.98 | |
| 34 | 180 | 0.2 | 0.05 | 1.52 | |
| 35 | 200 | 0.1 | 0.025 | 1.61 | |
| 36 | 200 | 0.1 | 0.04 | 1.59 | |
| 37 | 150 | 0.1 | 0.025 | 2.45 | |
| 38 | 150 | 0.1 | 0.0325 | 2.46 | |
| 39 | 150 | 0.1 | 0.05 | 2.26 | |
| 40 | 160 | 0.2 | 0.05 | 2.53 | |
| 41 | 160 | 0.25 | 0.04 | 2.42 | |
| 42 | 180 | 0.15 | 0.04 | 1.90 | |
| 43 | 180 | 0.25 | 0.025 | 2.32 | |
| 44 | 200 | 0.15 | 0.025 | 1.80 | |
| 45 | 200 | 0.2 | 0.0325 | 2.41 | |
| 46 | 150 | 0.15 | 0.0325 | 2.00 | |
| 47 | 150 | 0.15 | 0.05 | 1.66 | |
| 48 | 150 | 0.2 | 0.025 | 2.23 | |
| 49 | 160 | 0.1 | 0.025 | 1.56 | |
| 50 | 160 | 0.1 | 0.05 | 1.66 | |
| 51 | 180 | 0.1 | 0.04 | 1.66 | |
| 52 | 180 | 0.25 | 0.025 | 1.83 | |
| 53 | 200 | 0.2 | 0.0325 | 1.55 | |
| 54 | 200 | 0.25 | 0.04 | 1.43 | |
| 55 | 150 | 0.15 | 0.025 | 1.92 | |
| 56 | 150 | 0.15 | 0.05 | 1.86 | |
| 57 | 150 | 0.2 | 0.04 | 1.53 | |
| 58 | 160 | 0.1 | 0.025 | 1.80 | |
| 59 | 160 | 0.1 | 0.04 | 1.77 | |
| 60 | 180 | 0.1 | 0.0325 | 1.66 | |
| 61 | 180 | 0.25 | 0.025 | 1.80 | |
| 62 | 200 | 0.2 | 0.05 | 1.23 | |
| 63 | 200 | 0.25 | 0.0325 | 1.46 | |
| 64 | 150 | 0.2 | 0.04 | 2.02 | |
| 65 | 150 | 0.25 | 0.025 | 2.18 | |
| 66 | 150 | 0.25 | 0.05 | 2.06 | |
| 67 | 160 | 0.15 | 0.0325 | 1.84 | |
| 68 | 160 | 0.2 | 0.025 | 2.10 | |
| 69 | 180 | 0.1 | 0.0325 | 1.95 | |
| 70 | 180 | 0.15 | 0.04 | 1.84 | |
| 71 | 200 | 0.1 | 0.025 | 1.80 | |
| 72 | 200 | 0.1 | 0.05 | 1.80 | |
| 73 | 150 | 0.1 | 0.025 | 2.76 | |
| 74 | 150 | 0.1 | 0.04 | 2.21 | |
| 75 | 150 | 0.1 | 0.05 | 2.22 | |
| 76 | 160 | 0.25 | 0.0325 | 1.17 | |
| 77 | 160 | 0.25 | 0.04 | 1.11 | |
| 78 | 180 | 0.2 | 0.025 | 1.37 | |
| 79 | 180 | 0.2 | 0.05 | 1.33 | |
| 80 | 200 | 0.15 | 0.025 | 1.52 | |
| 81 | 200 | 0.15 | 0.0325 | 1.21 | |
